# Transient Postpartum Paraparesis Mimicking Guillain–Barré Syndrome Following Labor Epidural Analgesia: A Case Report

**DOI:** 10.7759/cureus.92314

**Published:** 2025-09-14

**Authors:** Ahmed Alanzi, Reem AlAamer, Dawood Alatefi, Bano Alsaleh, Ahmed M Hussain, Samah Hakmi

**Affiliations:** 1 Anesthesia and Pain Management, King Hamad University Hospital, Muharraq, BHR; 2 Neurosurgery, Center for Spinal Surgery, Werner-Wicker-Clinic, Bad Wildungen, DEU; 3 Radiology, King Hamad University Hospital, Muharraq, BHR; 4 Anesthesia and Pain Management Department, King Hamad University Hospital, Muharraq, BHR

**Keywords:** cesarean section, epidural analgesia complications, guillain–barré syndrome, mri, postpartum paraparesis, transient neurological deficit

## Abstract

Transient postpartum neurological deficits are rare but potentially alarming, sometimes mimicking conditions such as Guillain-Barré syndrome (GBS) and necessitating urgent evaluation to exclude structural, infectious, or inflammatory causes. We report the case of a 25-year-old primigravida with alpha-thalassemia trait who presented at 40+1 weeks for spontaneous labor. She received epidural analgesia without immediate complications but subsequently required emergency cesarean section under general anesthesia for failure to progress. On postoperative day two, she developed bilateral lower-limb numbness, heaviness, and weakness that progressed within hours to quadriparesis. Neurological examination initially showed preserved upper-limb strength with lower-limb weakness, then evolved to reduced motor power in both upper and lower limbs, diminished sensation below T6, and preserved anal tone. The clinical picture raised suspicion for GBS, and she was admitted to the ICU, where her respiratory status was closely monitored; no ventilatory compromise occurred. Extensive workup, including urgent MRI of the whole spine and cerebrospinal fluid analysis on the same day, along with nerve conduction studies within 24 hours, was unremarkable. By postoperative day three, neurological function began to improve spontaneously without specific intervention; full recovery was achieved by postoperative day eight, with no residual deficits on follow-up. This case underscores the importance of a comprehensive, multidisciplinary approach (neurology, neurosurgery, orthopedics, obstetrics, anesthesiology, and intensive care) when evaluating postpartum neurological symptoms. The rapid spontaneous recovery without intervention suggests a transient, self-limiting neurological event, possibly due to neuraxial inflammation or ischemia.

## Introduction

Paraparesis, defined as bilateral lower-limb weakness with varying degrees of sensory or autonomic involvement, is a rare and distressing complication in the postpartum period. Although uncommon, with an estimated incidence of fewer than 1 in 10,000 deliveries [1], its occurrence poses significant diagnostic and management challenges for clinicians. In the setting of labor and delivery, particularly after regional anesthesia or cesarean section, sudden neurological deficits necessitate urgent differentiation between obstetric, anesthetic, and neurological causes, since delays in recognition and treatment may result in permanent deficits. Important differential diagnoses include epidural hematoma, spinal abscess, direct spinal cord injury, and, less commonly, acute inflammatory demyelinating polyneuropathies such as Guillain-Barré syndrome (GBS) [2].

GBS is a well-recognized cause of postpartum neurological deterioration, but its presentation may overlap with obstetric or anesthetic complications, particularly when progressive weakness, sensory loss, or autonomic dysfunction occurs in the days following delivery [3,4]. Transient postpartum neurological deficits, on the other hand, remain sparsely documented. Imaging and laboratory investigations are frequently normal, leaving diagnosis primarily clinical and retrospective. Management in such cases is largely supportive, with urgent exclusion of structural causes and early involvement of a multidisciplinary team including anesthesiology, neurology, neurosurgery, obstetrics, and intensive care considered essential to optimize patient outcomes [5-7].

Here, we report a novel case of transient postpartum paraparesis that progressed to quadriparesis but resolved completely within days without immunotherapy or surgical intervention. This combination of unusual progression and rapid spontaneous recovery distinguishes our case from previously reported postpartum paraparesis and GBS cases.

## Case presentation

A 25-year-old primigravida woman with known alpha-thalassemia trait presented to the labor and delivery emergency department at 40 weeks and one day of gestation with lower abdominal pain due to the spontaneous onset of labor contractions. She requested epidural analgesia and was counseled regarding the procedure, including risks, benefits, and potential complications such as post-dural puncture headache.

At the time of epidural placement, the patient was in the active phase of the first stage of labor, with regular contractions and cervical dilation of 4 cm. Epidural analgesia was performed in the sitting position under strict aseptic precautions. A 16-G Tuohy needle was inserted at the L3-L4 interspace using the loss-of-resistance to saline technique, and a catheter was advanced 3-5 cm into the epidural space and secured. A test dose of 3 mL of 1.5% lidocaine with 1:200,000 epinephrine was administered to exclude intrathecal or intravascular placement. Following a negative response, a bolus of 10 mL of 0.1% bupivacaine with fentanyl 2 mcg/mL was given, and an infusion of 5 mL/hr of 0.08% bupivacaine with fentanyl 2 mcg/mL was commenced via infusion pump. The patient was repositioned supine and monitored continuously.

Due to failure to progress in labor, she underwent an emergency cesarean section (EMCS) under general anesthesia. Although an epidural catheter was in situ, it had been used exclusively for labor analgesia and did not provide an adequate block for surgical anesthesia. Given the urgency of delivery, general anesthesia was chosen to ensure rapid and reliable anesthetic conditions. Postoperatively, as the catheter had not been utilized intraoperatively and adequate systemic analgesia was achieved, it was removed in accordance with institutional practice to minimize the risk of infection; the tip was confirmed intact. She was then transferred to the post-anesthesia care unit (PACU), where she met discharge criteria and was subsequently returned to the ward.

On postoperative day two, the patient developed bilateral lower-limb numbness and heaviness associated with difficulty walking and sitting, in addition to localized pain at the surgical site. Symptoms began around midday, briefly improved with analgesia, but recurred later the same day. She denied trauma, falls, urinary retention, or bowel incontinence. While no dorsal spinal tenderness was documented, localized tenderness was noted over the upper lumbar region near the epidural insertion site.

Neurological examination showed preserved motor strength in the upper limbs, but significant bilateral lower-limb weakness with associated sensory impairment below T6. Deep tendon reflexes were brisk at the knees and ankles bilaterally. Hoffmann’s sign was positive bilaterally [6]. Babinski reflexes were downgoing, with no ankle clonus [7]. Based on these findings, the patient was classified as ASIA grade D, with a neurological level at T6 [8]. A detailed summary of the neurological examination is presented in Table 1.

**Table 1:** Neurological Examination Findings on Postoperative Day 2 Abnormal findings are highlighted in bold

PARAMETER	RIGHT SIDE	LEFT SIDE	NOTES
Motor Power (MRC scale)	L2: 3/5, L3: 3/5, L4: 0/5, L5: 0/5, S1: 3/5	L2: 3/5, L3: 4/5, L4: 0/5, L5: 0/5, S1: 3/5	Upper limbs: 5/5 (normal)
Sensory Level	Diminished below T6; absent pinprick T10–T12	Diminished below T6; absent pinprick T10–T12	Light-touch & pinprick ↓
Reflexes	Knee jerk: brisk; Ankle jerk: brisk	Knee jerk: brisk; Ankle jerk: brisk	Upper-limb reflexes intact
Pathological Reflexes	Hoffmann’s: positive; Babinski: downgoing	Hoffmann’s: positive; Babinski: downgoing	No clonus
Anal Tone	Preserved	Preserved	Sensation ↓ perianal
ASIA Grade	–	–	D (neurological level T6)

Over the following hours, her condition worsened to quadriparesis, with painful stimuli eliciting only minimal flickering movements in all four limbs. The rapid progression raised strong suspicion for GBS or another acute demyelinating polyneuropathy.

Multidisciplinary consultations with orthopedics, neurosurgery, neurology, intensive care, and obstetrics were arranged. The patient and her family were counseled regarding the critical condition, the risk of respiratory compromise, and the potential need for ICU admission with intubation and invasive ventilation. She was transferred to the ICU for monitoring and further workup.

**Figure 1:**
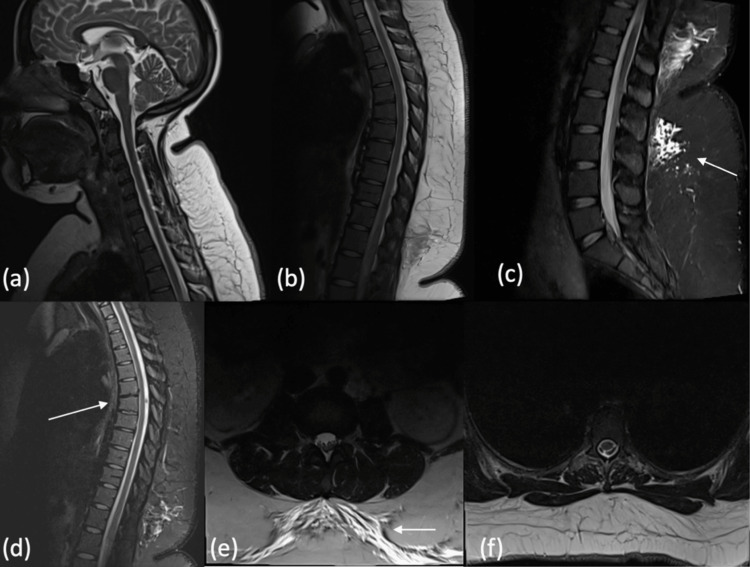
Whole Spine MRI Without Contrast (a) sagittal cervical spine with preserved alignment, canal diameter, and cord signal; (b) sagittal thoracic spine with no abnormal findings; (c) sagittal lumbar spine with no abnormal findings; (d) mild anterior wedging of the D6 vertebra; (e) small areas of lumbar subcutaneous edema, likely related to epidural puncture; (f) axial lumbar section showing preserved cord signal and absence of compression.

Routine laboratory investigations were within normal limits. Plain radiographs of the lower and dorsal spine were unremarkable. Urgent MRI of the entire spine demonstrated no compressive or structural pathology, with preserved vertebral alignment, canal diameter, and cord signal. Only mild anterior wedging of the D6 vertebra and small areas of lumbar subcutaneous edema (likely related to epidural puncture) were observed (Figure 1). 

**Figure 2:**
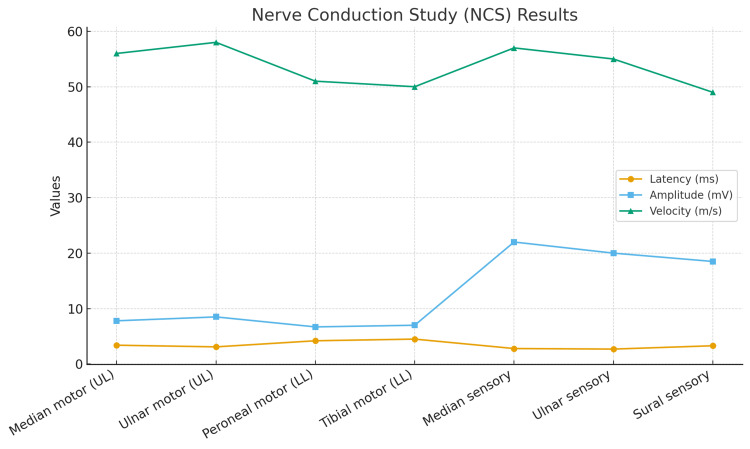
Nerve Conduction Study (NCS) Findings

A lumbar puncture was performed under strict aseptic conditions with a 22-G Quincke needle. Cerebrospinal fluid (CSF) analysis (Table 2) demonstrated clear appearance, normal cell count, protein, and glucose values, effectively excluding infectious or inflammatory processes. Nerve conduction studies (Figure 2) revealed normal motor and sensory responses in both upper and lower limbs, with preserved latencies, amplitudes, and conduction velocities, providing no evidence of demyelination or axonal neuropathy.

**Table 2:** Cerebrospinal Fluid (CSF) Analysis Findings

PARAMETER	RESULT	REFERENCE RANGE	INTERPRETATION
Appearance	Clear	Clear	Normal
Cell count	2 cells/µL	<5 cells/µL	Normal
Protein	34 mg/dL	15–45 mg/dL	Normal
Glucose (CSF)	62 mg/dL	40–70 mg/dL	Normal
Simultaneous blood glucose	95 mg/dL	—	CSF:serum ratio >0.5 → Normal

During her ICU stay, the patient was assessed daily by the neurology team, who advised close monitoring without initiation of immunotherapy in view of normal CSF and NCS findings. Supportive management included multimodal analgesia, physiotherapy, and early mobilization

By postoperative day three, the patient began to show improvement. Upper-limb power (C5-T1) was fully restored, with residual grade 2 sensory reduction. Lower-limb motor strength (L2-S1) was also full bilaterally, with mild persistent sensory reduction. Reflexes were symmetrical, Hoffman’s sign was negative, Babinski’s downgoing, and no clonus was present.

She remained hemodynamically stable, afebrile, and able to mobilize independently, allowing transfer from the ICU to the ward. By postoperative day eight, she met discharge criteria and was discharged home. At follow-up with the pain management team, she walked independently, reported no weakness or numbness, and had a normal neurological examination. Inspection of the epidural insertion site showed no evidence of infection, hematoma, or local complications. She was discharged from further follow-up. The clinical course and diagnostic workflow are summarized in the Appendix.

## Discussion

In this case, suspicion of GBS arose when the patient developed acute bilateral lower-limb weakness and sensory loss progressing to quadriparesis on postoperative day two. However, her neurological deficits improved spontaneously over day three without specific treatment (neurological findings summarized in Table 1). Although GBS was initially considered, normal cerebrospinal fluid (CSF) findings, normal nerve conduction studies, and full recovery without immunotherapy ultimately made the diagnosis unlikely.

Postpartum neurological deficits present a diagnostic challenge, particularly in patients who have received neuraxial anesthesia. The differential diagnosis ranges from life-threatening causes to benign transient conditions. GBS is an acute, immune-mediated polyradiculoneuropathy that typically presents with rapidly ascending, symmetrical weakness and areflexia [9]. CSF analysis usually reveals albuminocytologic dissociation, characterized by elevated protein with normal cell count [10]. Pregnancy and the postpartum period are recognized triggers for GBS, likely related to immune reconstitution after delivery [11].

For example, Sharma et al. studied 47 patients with pregnancy-associated GBS and found the risk to be highest during the third trimester and within two weeks postpartum [12]. Several reports have described postpartum GBS [13]. The condition may worsen in this period because of delayed-type hypersensitivity responses. During pregnancy, immune balance shifts toward Th2 dominance and increased regulatory T-cell activity, particularly in early trimesters. GBS, however, is largely associated with Th1-mediated mechanisms. Although this shift does not fully explain all immunological changes observed, it contributes to the understanding of disease modulation [14]. Inflammatory cytokines such as IFN-γ, TNF-α, IL-6, and IL-1 have demonstrated protective roles against GBS, although the precise pathways remain unclear [15].

In our patient, GBS was considered unlikely because CSF protein and nerve conduction studies were normal, and symptoms resolved without immunoglobulin therapy. Furthermore, weakness in GBS typically peaks over days to weeks [16], whereas our patient recovered dramatically within a day. It is important to acknowledge, however, that very early GBS can occasionally present with initially normal investigations. In this context, the rapid and complete spontaneous recovery observed in our patient remains the most reliable distinguishing feature, making GBS an improbable diagnosis.

It should also be emphasized that the epidural catheter, although in situ, had been used exclusively for labor analgesia and was not providing a reliable block for surgical anesthesia. Given the urgency of delivery, general anesthesia was therefore chosen to ensure rapid and effective anesthetic conditions. The catheter was subsequently removed postoperatively in accordance with institutional practice to minimize infection risk, as it was no longer required for analgesia.

Another important differential is spinal epidural hematoma, a rare but serious complication of neuraxial blockade. It usually presents within hours of epidural placement or catheter removal, causing severe back pain and rapidly progressive paralysis [17]. In our patient, the epidural catheter was removed on the same day as the cesarean section because it had not been used intraoperatively and adequate systemic analgesia was achieved postoperatively; this reflects our institutional practice of removing unused catheters promptly to minimize infection risk. Importantly, her coagulation profile was normal before surgery, further lowering the likelihood of catheter-related hematoma formation. Spinal cord infarction is another consideration, often causing acute paraplegia with sudden onset and linked to hypotension, aortic injury, or embolism. Rarely, epidural anesthesia has been implicated in precipitating cord ischemia through hypotension, vasospasm, or local anesthetic toxicity [18]. In this case, MRI showed no infarct, and vertebral alignment was normal.

Other conditions to exclude include transient neuraxial block complications and functional neurological disorder. Transient neurological deficits after neuraxial anesthesia have been reported, potentially due to unusually high cephalad spread of local anesthetic, neurotoxicity, or nerve root irritation from catheter trauma. For instance, Prashanth et al. described a parturient who developed flaccid paraplegia after a labor epidural and recovered fully after catheter removal [19].

The exact mechanism of transient paraparesis in our case remains unclear, though several hypotheses can be considered. One possibility is a transient inflammatory or ischemic process affecting spinal nerve roots or cord segments, as epidural catheter placement may provoke local inflammation or vascular spasm [19]. Such vasospasm or hypoperfusion could produce symptoms without permanent imaging abnormalities. Another consideration is local anesthetic neurotoxicity; however, this complication is typically associated with higher concentrations of agents, particularly lidocaine [20]. In our case, a low-concentration bupivacaine with fentanyl regimen was used, which is generally considered safe, making direct neurotoxicity unlikely. This supports the interpretation that transient ischemic or inflammatory changes were the more plausible underlying mechanism.

Overall, a thorough multidisciplinary evaluation, including urgent MRI and CSF analysis, is essential in any case of acute postpartum neurological deficit following neuraxial anesthesia [17]. Despite a rapid recovery in our patient, no definitive etiology was identified, leaving some uncertainty. While an early nerve conduction study may theoretically miss mild demyelination, the clinical course argues strongly against GBS.

## Conclusions

This case highlights a rare but significant diagnostic challenge in the postpartum period. The patient’s rapid progression from paraparesis to quadriparesis, with preserved reflexes and sensory changes, closely mimicked GBS. However, extensive diagnostic workup, including MRI, CSF analysis, and nerve conduction study, was unremarkable, and she achieved complete spontaneous neurological recovery without immunotherapy or surgical intervention. The novelty of this case lies in the unusual progression and dramatic recovery, distinguishing it from previously reported postpartum GBS or neuraxial complications. The key clinical learning point is that transient postpartum quadriparesis can mimic GBS yet resolve spontaneously. While the prognosis here was favorable, such presentations necessitate early ICU monitoring and prompt multidisciplinary involvement to ensure patient safety and optimize outcomes.

## Figures and Tables

**Figure 3:**
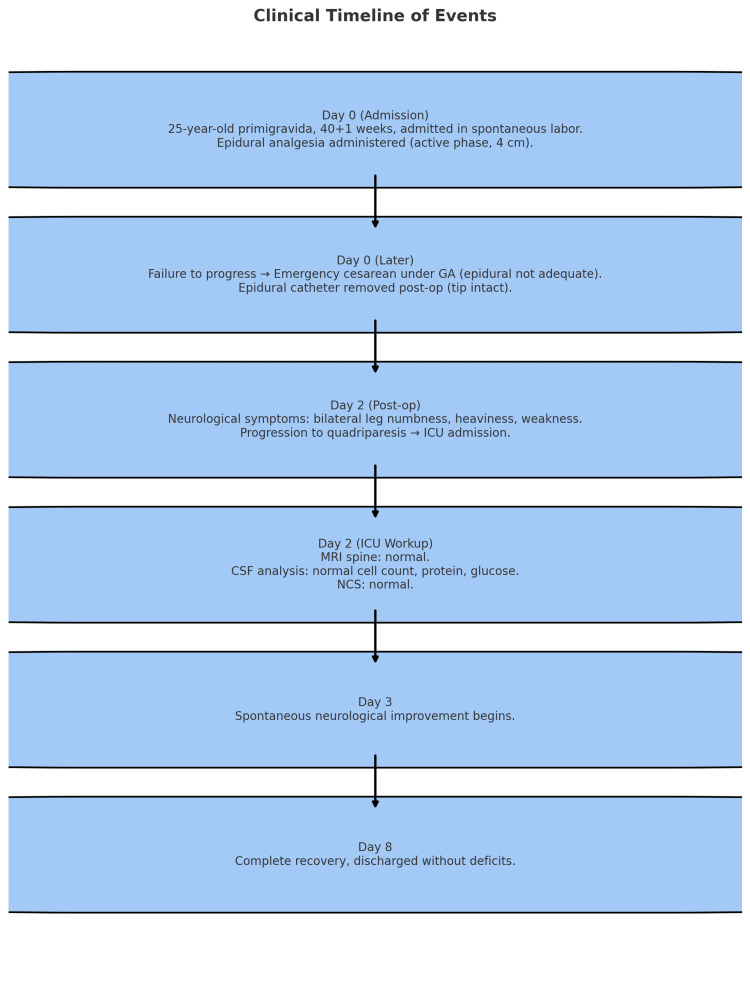
Diagnostic Workflow

## Appendices

Appendix 
